# Supplementary material for: Probing atomic physics at ultrahigh pressure using laser-driven implosions
Source: Nat Commun. 2022 Nov 16;13:6780. doi: 10.1038/s41467-022-34618-6 (PMC9668816; doi:10.1038/s41467-022-34618-6)
Supplement: Supplementary file 1 — Supplementary Information [file 41467_2022_34618_MOESM1_ESM.pdf]

Supplementary Information

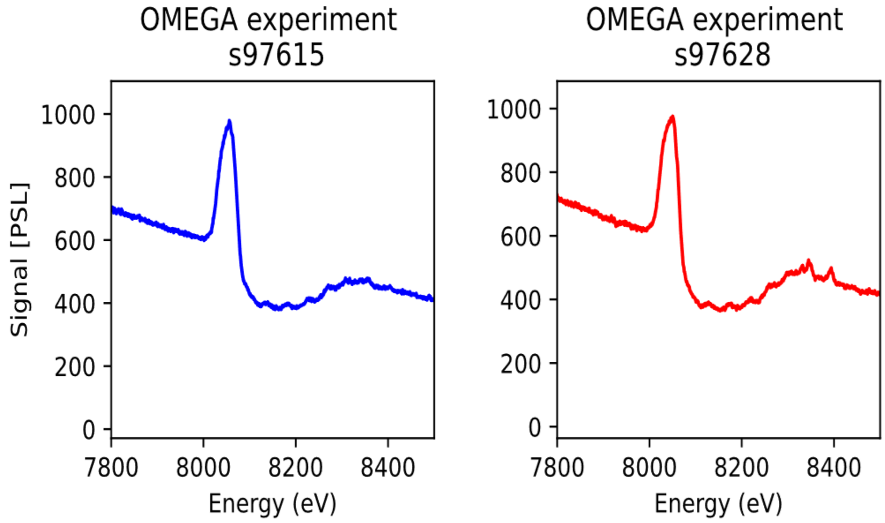

Supplementary Figure 1: Demonstration of the experimental reproducibility for implosion spectroscopy on OMEGA. The spectra shown here are from separate implosions with nominally identical laser pulse-shapes and targets as discussed in the paper.

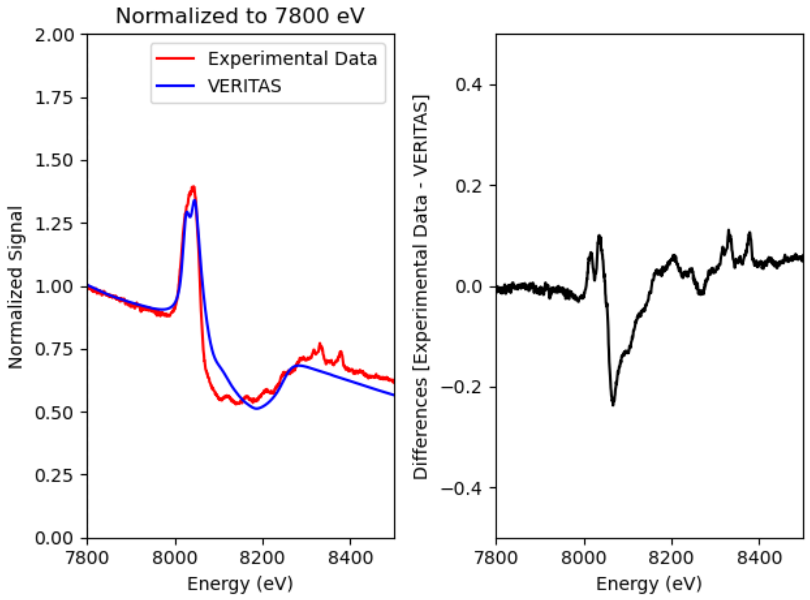

Supplementary Figure 2: Overlay of experimental data and *VERITAS* simulation of time-integrated x-ray spectra (left) and the corresponding residual plot (right).

Supplementary Note 1. EXPERIMENTAL REPRODUCIBITY

These implosion x-ray spectroscopy experimental campaigns have been conducted over three years on OMEGA. Multiple nominally identical shots have been conducted. Neutron measurements, time-gated images, and time-integrated spectra recorded on such high-adiabat and relatively-lower implosion velocity experiments indicate a high degree of repeatability. Supplementary figure 1 shows the time-integrated x-ray spectra measured for two such repeatable shots. For these spherical implosion spectroscopy campaigns on OMEGA, we have scanned some key experimental parameters, including laser pulse shape (1ns and 2-ns square pulse), the distance of the Cu-dopant layer from the inner surface of the shell (3 to 10 μm), and the concentration of Cu (2% to 4%). All of such high-adiabat and relatively-lower velocity implosions are robust and repeatable. Detailed descriptions of experimental parameter scans and their comparisons to *VERITAS* modeling will be reported elsewhere.

Supplementary Note 2. RESIDUAL PLOT

Supplementary figure 2 shows an overlay of experimental data and the results of a *VERITAS* simulation (left panel) and the corresponding residual plot (right panel). As stated in the main text, the signals are normalized at the photon energy of 7800 eV. The residual plot shows that the overall difference between experiment and *VERITAS* is within ~0.1 of the normalized experimental values, except for the regime of hν ≈ 8070 eV in which the difference approaches ~0.23. This localized and large difference, appearing during the return shock heating, might be attributed to the uncertainty of broadening treatment currently adopted in *VERITAS* from other models. In addition, a small difference in slope between the measured and simulated continuum emission can be seen in Supplementary figure 2. This difference likely comes from uncertainty in the stagnation plasma conditions, particularly the difference between the experimental hot-spot temperature and that from radiation-hydrodynamic simulations (see Table II of main text).
